# Squeeze-and-Excitation Enhanced Convolutional Neural Networks for Multi-class Pneumonia Classification on Chest Radiographs

**DOI:** 10.7759/cureus.99649

**Published:** 2025-12-19

**Authors:** Kian A Huang, Haris K Choudhary, Ashley Santiago, Neelesh S Prakash

**Affiliations:** 1 Radiology, University of South Florida Morsani College of Medicine, Tampa, USA

**Keywords:** artificial intelligence, chest x-ray, convolutional neural networks, covid-19, deep learning, machine learning, medical imaging, pneumonia, squeeze-and-excitation, transfer learning

## Abstract

This study compared two convolutional neural network (CNN) architectures, ResNet50V2 and InceptionV3, each enhanced with squeeze-and-excitation (SE) attention mechanisms, for automated classification of chest X-rays into normal, pneumonia-bacterial, pneumonia-viral, and COVID-19 categories. A total of 9,208 posterior-anterior chest radiographs were analyzed, divided into training, validation, and test datasets under identical preprocessing and fine-tuning conditions.

ResNet50V2-SE achieved a test accuracy of 98.18% and a macro-averaged area under the curve (AUC) of 0.9951, while InceptionV3-SE achieved 97.86% accuracy and an AUC of 0.9949. Class-specific evaluation showed that ResNet50V2-SE classified normal radiographs with perfect precision, recall, and F1-score (1.00). Pneumonia-bacterial images were classified with a precision, recall, and F1-score of 0.99, while pneumonia-viral and COVID-19 images reached F1-scores of 0.98 and 0.95, respectively. Comparable results were observed with InceptionV3-SE, achieving F1-scores of 1.00 for normal, 0.99 for pneumonia-bacterial, 0.97 for pneumonia-viral, and 0.95 for COVID-19.

McNemar's test was performed to compare model performance on the same test set. ResNet50V2-SE was correct in eight cases where InceptionV3-SE was incorrect, whereas InceptionV3-SE was correct in two cases where ResNet50V2-SE was incorrect. The resulting χ² statistic was 0.1250 (p>0.05), indicating no statistically significant difference in classification performance between the two models.

Both SE-enhanced CNN architectures demonstrated high accuracy and generalizability for differentiating among pneumonia subtypes on chest X-rays. These findings suggest that attention-augmented deep learning models may serve as effective decision-support tools in radiologic diagnosis, warranting further validation across larger and more diverse clinical datasets.

## Introduction

Between 2019 and 2021, there were approximately 489 million cases of pneumonia worldwide with an estimated total mortality rate of 0.5% [1]. In 2023, there were 41,210 deaths due to pneumonia in the US alone, equaling 12.3 average deaths per 100,000 [2]. Pneumonia is a respiratory infection that can be classified by the type of microorganism causing it: bacterial, viral, or fungal [1]. Studies indicate that evidence of viral infection ranges between 43% to 67% of cases in pneumonia patients, suggesting that viral pneumonia accounts for approximately half of all pneumonia cases, with bacterial pneumonia representing the other major category [3]. 

Diagnosing pneumonia generally requires the visualization of infiltrate on chest imaging in a patient presenting with symptoms including fever, dyspnea, cough, and sputum production [4]. Notable findings that may constitute a pneumonia diagnosis include lobar consolidations, interstitial infiltrates, and cavitations. Although useful for diagnosis, radiographs on their own cannot always reliably differentiate between different etiologies and pathogenesis of pneumonia, and their interpretation is prone to subjective variability. Because the radiographic findings with pneumonia are nonspecific, have little distinction between etiologies, and are subject to variable interpretation by experienced radiologists, clinicians must still keep a wide set of differential causes when pneumonia is suspected [4]. This discrepancy provides an opportunity for modern artificial intelligence (AI) approaches to be used in pneumonia diagnosis and classification. 

AI refers to the use of computers and machines to learn from and adapt to new or past experiences, as well as performing complex problem-solving and decision-making.

Convolutional Neural Networks (CNNs) represent a specialized class of AI deep learning algorithms particularly well-suited for image analysis [5]. CNNs utilize convolutional layers when extracting features from given inputs, and subsequently pool the layers together to reach a classification [5]. Because CNNs mimic the complex pathways that allow human brains to process visual stimuli, these deep learning algorithms are particularly effective for analyzing and interpreting visual data [5-7]. CNNs have demonstrated strengths in capturing detailed textural and morphological patterns that are crucial to identifying pneumonia signs [8-12].

Recent studies have demonstrated the use of CNN models for pneumonia detection by using chest radiographs across various patient populations. For example, Rajpurkar et al.'s CheXNet model utilized a DenseNet121 architecture on a large adult chest X-ray dataset to assess fourteen different pathologies, and it was able to achieve an area under the curve (AUC) of 0.851 for detecting pneumonia, which was comparable to that of radiologists, which underscores CNN's clinical potential in adult cohorts [12]. 

Similarly, in pediatric populations, CNNs such as VGG-19 have been applied successfully, with Saboo et al. reporting 87.9% accuracy in differentiating bacterial and viral pneumonia from normal chest X-rays in children [13]. Furthermore, Mujahid et al. tested multiple CNN models, including InceptionV3, ResNet50, and VGG-16, on a pediatric chest X-ray dataset and demonstrated that ensemble models were able to obtain accuracies of 99.29%, 98.93%, and 98.06%, respectively, for distinguishing normal and pneumonia cases [14]. These studies collectively highlight the growing effectiveness of CNN-based approaches enhanced by transfer learning for pneumonia diagnosis in both adult and pediatric settings.

The CNN models that will be used in this study and have had previous applications include the ResNet50V2 and InceptionV3 [5,14]. ResNet50V2 is a CNN architecture composed of 50 layers that is designed to effectively learn image features by addressing the significant challenge of the vanishing gradient problem in deep learning. This model introduces the skip connections that allow information to bypass one or more layers, which allows the model to learn residual functions instead of attempting to learn the full desired mapping, leading to performance degradation. This approach allows for more stable and efficient training of very deep networks, making ResNet50V2 particularly suitable for complex medical imaging tasks that require fine-grained feature discrimination, such as tumor classification [15]. For brain tumor classification, ResNet50V2 was able to achieve 92.6% accuracy in MRI-based tumor detection [5].

InceptionV3 is a CNN architecture that optimizes image feature extraction by efficiently capturing information at multiple spatial scales simultaneously. Unlike other CNN models, InceptionV3 utilizes inception modules that apply parallel convolutional filters of varying sizes on the inputs, which allows the network to capture both fine-grained details and broader contextual patterns within the same layer. This architectural design allows InceptionV3 to achieve a balance between accuracy and computational cost, making it highly effective for complex image classification tasks in medical imaging. As mentioned previously, the InceptionV3 model achieved the highest accuracy among the models in identifying pneumonia on chest radiographs (albeit only using a binary classification between healthy and diseased radiographs), with a 99.29% accuracy score, and 98.83%, 99.73%, and 99.28% scores for precision, recall, and F1, respectively [14]. Overall, these models mainly differ in their method for feature extraction, with ResNet50V2 utilizing skip connections for stable learning of hierarchical features, while InceptionV3 employs parallel multi-scale convolutions for simultaneous capture of features at different resolutions.

As explained, while CNN excels at automatic feature extraction, this leads to the architectures treating all feature outputs or feature channels equally, which leads to a lack of mechanisms to prioritize the most diagnostically relevant information. Additionally, CNNs prioritize localized spatial details while overlooking the integration of broader contextual patterns that could enhance feature refinement. This limitation can be especially problematic in medical imaging, where subtle pathological changes may be overshadowed by irrelevant background information or anatomical variations. Attention mechanisms can address this limitation by improving CNN performance by selectively emphasizing important features and capturing complex interdependencies, while also considering the impact of less relevant information. By selectively emphasizing spatial, channel-wise, or combined feature representations, attention mechanisms enhance model efficiency, adaptability, and generalization in tasks like image classification [16].

Previous research has shown that squeeze-and-excitation (SE) attention frameworks confer superior classification performance compared to other attention strategies [7]. It is the reason the SE mechanism will be utilized in this study. SE blocks can enhance a CNN through a computationally efficient two-step attention mechanism. First, the squeeze operation aggregates the spatial information across each of the feature maps to model long-range dependencies across the image. Second, the excitation operation applies channel-wise weights, which allows the network to emphasize informative features and suppress less relevant ones. Unlike traditional CNNs with fixed static filter responses, SE blocks dynamically recalibrate feature importance based on the specific input, thereby improving the network's ability to represent complex patterns and boosting performance in visual recognition tasks [5,7,16,17].

This current study demonstrates the comparison of two high-performing CNN backbones, InceptionV3 and ResNet50V2, both augmented with SE blocks, and fine-tuned under identical preprocessing, optimization, and evaluation pipelines. The experimental framework examines the feasibility of two common architectures for image classification in the context of multi-class chest X-ray classification.

## Materials and methods

This study utilized a publicly available chest X-ray dataset curated from Mendeley Data, which included a total of 9,208 adults and pediatric posterior-anterior (PA) radiographic chest images. The dataset comprised four diagnostic categories: COVID-19 (n=1,281), normal (n=3,270), pneumonia-bacterial (n=3,001), and pneumonia-viral (n=1,656). All images were stored in a single directory and labeled by folder name. To ensure consistent preprocessing, images were filtered to include common formats such as .jpg, .png, and .bmp. The dataset was randomly split into training (70%, n=6,446), validation (15%, n=1,381), and test (15%, n=1,381) subsets using train_test_split from scikit-learn with a fixed random seed of 42 to preserve reproducibility. Class distributions were stratified across each subset to maintain proportional representation of all four categories. Prior to model training, all images were resized to 299×299 pixels to meet the input requirements of the InceptionV3 architecture and 224x224 pixels for ResNet50V2. Pixel values were normalized using the preprocess_input function corresponding to each model backbone. No explicit data augmentations were applied during this baseline comparative analysis due to the inherent stability of chest X-rays, allowing a direct assessment of architectural performance without augmentation bias.

Two convolutional neural network architectures were implemented and evaluated: InceptionV3 and ResNet50V2, each augmented with an SE block. The InceptionV3 model, pretrained on ImageNet and excluding its top classification layers, utilizes parallel branches of varying convolutional kernel sizes (1×1, 3×3, 5×5) and max pooling to capture multi-scale spatial features. To enhance the network's ability to recalibrate channel-wise feature responses, an SE block was integrated after the final inception module. The SE block applied global average pooling followed by two dense layers with a reduction ratio of 16 (rectified linear unit (ReLU) followed by Sigmoid activations), which generated channel-wise weights to rescale feature maps. This recalibration step helped the network emphasize more informative features relevant to disease classification.

In parallel, a ResNet50V2 architecture was implemented to compare residual-based feature extraction. ResNet50V2 introduces pre-activation residual bottleneck blocks and identity mappings, allowing for stable deep training through shortcut connections. Similar to the InceptionV3 configuration, an SE block was inserted after the final convolutional output layer in ResNet50V2, employing the same global pooling and excitation mechanism to recalibrate channel features. Both architectures shared a common classification head consisting of a GlobalAveragePooling2D layer followed by a fully connected stack: a dense layer with 512 units (ReLU activation), batch normalization, and dropout (rate=0.5); a subsequent dense layer with 128 units, batch normalization, and dropout (rate=0.3); and a final dense output layer with four neurons and softmax activation to predict one of the four diagnostic classes. L2 regularization (λ=1e−4) was applied to all dense layers to reduce overfitting.

For training, both models initially had their base layers frozen to preserve pretrained features. Fine-tuning was subsequently performed by unfreezing the final 50 layers in InceptionV3 and the final 30 layers in ResNet50V2. All models were compiled using the Adam optimizer with an initial learning rate of 1e−4. The categorical cross-entropy loss function was used in conjunction with label smoothing (α=0.1) to reduce model overconfidence and enhance generalization. Training proceeded with a batch size of 16 for up to 100 epochs. To prevent overfitting and reduce training time, early stopping was applied with a patience of seven epochs, while learning rate reduction on plateau (factor=0.5, patience=3, minimum learning rate=1e−7) allowed dynamic adjustment of the learning rate during stagnation.

Evaluation was conducted on both the validation and test datasets using a set of standard classification metrics to comprehensively assess model performance. Accuracy was used to quantify the overall proportion of correctly classified instances across all categories. Precision, defined as the proportion of true positives among all positive predictions, evaluates the model's ability to avoid false positives. Recall, or sensitivity, measures the proportion of true positives correctly identified out of all actual positive cases, thereby assessing the model's ability to detect relevant instances. The F1-score, calculated as the harmonic mean of precision and recall, provides a balanced metric that accounts for both types of classification errors. In addition to these discrete metrics, the area under the receiver operating characteristic curve (AUC) was computed for each class as well as across all classes using a macro-averaged approach. AUC reflects the model's ability to discriminate between classes across varying decision thresholds, with values closer to 1.0 indicating superior discriminatory capability. A confusion matrix was also generated to visualize the distribution of true versus predicted labels, enabling further analysis of class-specific misclassification patterns. Together, these metrics offer a multidimensional evaluation of diagnostic performance across all disease categories.

McNemar's test was performed to compare the classification performance of ResNet50V2-SE and InceptionV3-SE models. McNemar's test is a paired non-parametric statistical test appropriate for comparing two classifiers evaluated on the same dataset. The test examines discordant pairs-instances where one model classifies correctly while the other does not - to determine whether observed performance differences are statistically significant. The test statistic was calculated using the formula χ²=(|n₀₁-n₁₀|-1)²/(n₀₁+n₁₀) with continuity correction, where n₀₁ represents cases correctly classified by ResNet50V2-SE but misclassified by InceptionV3-SE, and n₁₀ represents the opposite scenario. Statistical significance was assessed at α=0.05.

## Results

**Figure 1:**
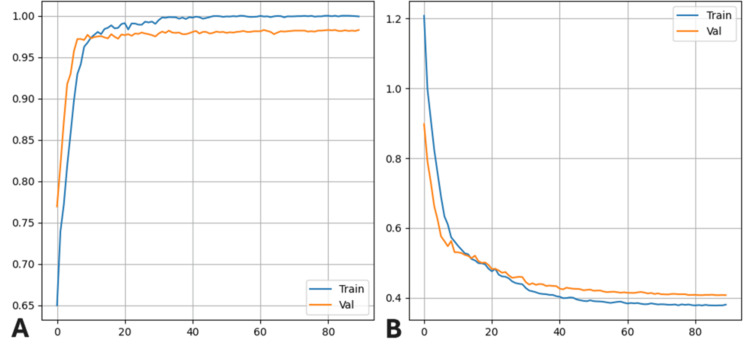
Training and validation performance curves of the ResNet50V2-SE model (A) Accuracy and (B) loss values over 90 training epochs. Both metrics demonstrate rapid convergence and stable generalization with minimal overfitting.

Both convolutional neural network models, ResNet50V2 with SE block and InceptionV3 with SE block, were trained on the same dataset and evaluated under identical conditions using the previously described train/validation/test split. Figure 1 illustrates the training and validation accuracy (A) and loss (B) curves for the ResNet50V2-SE model across epochs. The model rapidly converged within the first 20 epochs, achieving stable high validation accuracy with minimal overfitting, as evidenced by the close alignment between training and validation curves. The InceptionV3 demonstrated virtually identical learning and loss patterns in training and validation as well. Table 1 compares the final test accuracy and macro-averaged AUC scores for both architectures. ResNet50V2-SE achieved a higher overall test accuracy (0.9818) and slightly greater AUC (0.9951) than InceptionV3-SE (accuracy=0.9786, AUC=0.9949). As shown in Table 2, the ResNet50V2-SE model correctly classified normal images with perfect scores across all major metrics (precision=1.00, recall=1.00, F1-score=1.00, AUC=0.9999) on a support size of 357 images. Pneumonia-bacterial cases (n=890) were also classified with high accuracy, achieving a precision of 0.99, recall of 0.99, F1-score of 0.99, and AUC of 0.9983. For neumonia-viral cases (n=827), the model reached a precision of 0.97, recall of 0.99, F1-score of 0.98, and AUC of 0.9968. Performance on COVID-19 (n=454) was slightly lower but still strong, with a precision of 0.97, recall of 0.93, F1-score of 0.95, and AUC of 0.9852.

**Table 1:** Final test accuracy per model Testing accuracy per model on holdout test set. AUC - area under the receiver operating curve

Model	Test accuracy	Overall AUC
ResNet50V2	0.9818	0.9951
InceptionV3	0.9786	0.9949

**Table 2:** ResNet50V2-SE pneumonia classification results Classification metrics based on the holdout test set for ResNet50V2-SE. AUC - area under the receiver operating curve

Classification	Precision	Recall	F1-score	Support	AUC
Normal	1.00	1.00	1.00	357	0.9999
Pneumonia-bacterial	0.99	0.99	0.99	890	0.9983
Pneumonia-viral	0.97	0.99	0.98	827	0.9968
Pneumonia-COVID-19	0.97	0.93	0.95	454	0.9852

As detailed in Table 3, the InceptionV3-SE model also classified the normal class (n=357) with perfect scores (precision=1.00, recall=1.00, F1-score=1.00, AUC=0.9989). Pneumonia-bacterial cases (n=890) achieved precision and recall of 0.99, F1-score of 0.99, and AUC of 0.9989. Pneumonia-viral cases (n=827) were classified with a precision of 0.96, recall of 0.99, F1-score of 0.97, and AUC of 0.9957. For COVID-19 (n=454), InceptionV3-SE achieved a precision of 0.96, a recall of 0.93, an F1-score of 0.95, and an AUC of 0.9830. The class-wise confusion matrices for both models are presented in Table 4. In both models, most predictions fell along the diagonal, indicating a high rate of correct classifications across all categories. ResNet50V2-SE showed slightly fewer off-diagonal misclassifications compared to InceptionV3-SE. Misclassification patterns in both models were primarily observed between the pneumonia-viral and COVID-19 categories. The normal and pneumonia-bacterial classes exhibited strong diagonal dominance with minimal confusion against other categories.

**Table 3:** InceptionV3-SE pneumonia classification results Classification metrics based on the holdout test set for InceptionV3-SE. AUC - area under the receiver operating curve

Classification	Precision	Recall	F1-score	Support	AUC
Normal	1.00	1.00	1.00	357	0.9989
Pneumonia-bacterial	0.99	0.99	0.99	890	0.9989
Pneumonia-viral	0.96	0.99	0.97	827	0.9957
Pneumonia-COVID-19	0.96	0.93	0.95	454	0.9830

**Table 4:** Confusion matrices Confusion matrices per model reflecting correct and incorrect predictions with respect to the ground truth during the holdout test set.

ResNet50V2					InceptionV3			
Classification	Normal	Pneumonia-bacterial	Pneumonia-viral	COVID-19	Normal	Pneumonia-bacterial	Pneumonia-viral	COVID-19
Normal	356	1	0	0	356	1	0	0
Pneumonia-bacterial	0	884	0	6	0	882	3	5
Pneumonia-viral	0	0	818	9	1	0	815	11
COVID-19	0	4	26	424	0	4	29	421

As shown in Table 5, ResNet50V2-SE correctly classified 2,480 of 2,528 test images (98.18%), while InceptionV3-SE correctly classified 2,472 images (97.86%), representing a difference of eight correctly classified cases. Analysis of discordant pairs revealed that ResNet50V2-SE was correct in eight cases where InceptionV3-SE was incorrect (n₀₁=8), while InceptionV3-SE was correct in only two cases where ResNet50V2-SE was incorrect (n₁₀=2). The McNemar's test yielded a χ² statistic of 0.1250 (p>0.05), indicating no statistically significant difference between the two models' classification performance.

**Table 5:** Statistical comparison of model performance using McNemar's test Table demonstrating the performance differences between ResNet50V2-SE and InceptionV3 using ResNet50V2-SE as the reference model. McNemar's test was used based on the results from the identical hold-out test set used for both models. ᵃn₀₁ - cases where ResNet50V2-SE correct and InceptionV3-SE incorrect ᵇn₁₀ - cases where InceptionV3-SE correct and ResNet50V2-SE incorrect

Metric	ResNet50V2-SE
Test accuracy (%)	98.18
Correct classifications	2,480
Total test samples	2,528
Discordant pairs (n₀₁)ᵃ	8
Discordant pairs (n₁₀)ᵇ	2
McNemar's χ² statistic	0.1250
p-value	>0.05

## Discussion

This study evaluated and compared the diagnostic performance of two CNN architectures, ResNet50V2 and InceptionV3, each enhanced with SE blocks, for the task of multi-class classification of chest X-rays into normal, pneumonia-bacterial, pneumonia-viral, and COVID-19 categories. Both models demonstrated strong classification ability across all classes, with test accuracies exceeding 97.5% and macro-averaged AUC values above 0.994. These findings confirm that SE-augmented deep learning architectures can effectively distinguish between common pulmonary conditions on chest radiographs, even in the absence of handcrafted image features or clinical metadata. While both architectures achieved nearly perfect classification of normal and bacterial-pneumonia images, performance on pneumonia-viral and COVID-19 classes was marginally lower, suggesting that intra-class visual variability and inter-class similarities remain a challenge in radiographic interpretation.

Despite architectural differences, the ResNet50V2-SE model slightly outperformed the InceptionV3-SE model in nearly all evaluation metrics. To determine whether this observed performance difference was statistically significant, McNemar's test was performed to compare the two models on the same test set. McNemar's test is a paired non-parametric statistical procedure that specifically evaluates whether two classifiers make systematically different errors when evaluated on identical samples, rather than simply comparing overall accuracy rates. The test yielded a χ² statistic of 0.1250 (p>0.05), indicating no statistically significant difference between the two architectures. With only 10 discordant pairs out of 2,528 test samples, eight cases where ResNet50V2-SE was correct, and InceptionV3-SE was incorrect, versus two cases where the opposite occurred - the models demonstrated nearly identical classification behavior. This finding confirms that while ResNet50V2-SE achieved marginally higher accuracy (98.18% vs. 97.86%), representing a difference of only eight additional correct predictions, this 0.32% improvement likely reflects natural variance rather than a meaningful performance advantage. The lack of statistical significance suggests that both SE-enhanced architectures perform comparably for pneumonia classification, and the choice between them could be based on other practical considerations, such as computational efficiency, inference speed, or deployment constraints, rather than discriminative capability alone.

The comparable performance of both architectures may reflect the benefits of residual learning and deeper gradient flow inherent to the ResNet architecture, as well as its ability to preserve high-level semantic features across convolutional layers [15]. Conversely, the InceptionV3-SE model, designed for multi-scale feature extraction, also performed robustly, indicating that both residual and multi-branch architectural strategies remain viable for medical image classification tasks [14].

These results are consistent with prior studies that have applied transfer learning and attention mechanisms to medical imaging [5-7]. Recent literature has demonstrated the utility of SE modules for enhancing performance in imaging-based diagnostics, such as brain and lung tumor classification, especially compared to other forms of attention mechanisms [5-7]. In this context, the present work extends the evidence base to include chest radiographs for pneumonia subclassification, underlining the versatility of SE-enhanced transfer learning pipelines.

With regards to previous research using ResNet50 and InceptionV3 models without SE-enhancement, Mujahid et al. have demonstrated high accuracies on pediatric chest X-rays (98-99%), albeit only on a simpler and less challenging two-way classification comparing between diseased and healthy chest radiographics without distinction of etiology (bacterial, viral, and COVID-19) [14].

When compared to other deep learning approaches for chest X-ray classification, the SE-enhanced models demonstrate competitive or superior performance across multiple evaluation metrics. Rahman et al. reported that DenseNet201 achieved classification accuracies of 98%, 97%, and 99% for normal vs. pneumonia, bacterial vs. viral pneumonia, and three-way classification tasks, respectively [8]. While these results are comparable to our findings, this study's models, namely ResNet50V2-SE, achieved slightly higher overall accuracy (0.9818) in the more challenging four-way classification task that includes COVID-19 differentiation.

In comparison to AlexNet-based approaches, Ibrahim et al. reported performance varied depending on the classification task complexity. For binary classifications, the model achieved accuracies ranging from 91.43% to 99.62%, with the highest performance observed in COVID-19 vs. normal classification (99.16% accuracy) [9]. However, performance declined significantly in more complex classification scenarios, with four-way classification achieving only 93.42% accuracy and 89.18% sensitivity [9]. The SE-enhanced models substantially outperformed these results, maintaining accuracy above 98% with ResNet50V2-SE even in the four-way classification task, while achieving superior sensitivity and specificity across all pneumonia subtypes.

Similarly, VGG-19 architectures have shown promise in pneumonia detection, with reported accuracies of approximately 95% for general pneumonia classification tasks [10]. However, both the SE-augmented ResNet50V2 and InceptionV3 models exceeded this performance level while simultaneously addressing the more complex challenge of multi-class differentiation among pneumonia subtypes and COVID-19. The superior performance of this study's models across multiple evaluation metrics suggests that SE-enhanced architectures represent a meaningful advancement over conventional CNN approaches for chest radiograph analysis, particularly in scenarios requiring fine-grained differentiation between similar pathological conditions.

Misclassifications in both models were most prominent between the Pneumonia-Viral and COVID-19 categories. This is likely attributable to the substantial radiographic overlap between these two conditions, both of which can present with bilateral ground-glass opacities, patchy infiltrates, and consolidations that lack distinct morphological boundaries [10,11]. Such overlap poses a known diagnostic challenge and may limit model performance in distinguishing between viral etiologies based solely on PA chest X-rays [11]. Although both models maintained high recall and precision across all categories, the slightly lower F1-scores for COVID-19 and pneumonia-viral suggest that future iterations may benefit from incorporating additional clinical context or lateral imaging views to improve differentiation between these phenotypes.

From a clinical perspective, the ability to accurately classify chest radiographs into multiple pneumonia subtypes offers substantial utility. In particular, rapid differentiation between bacterial and viral pneumonia could inform early antibiotic stewardship, while reliable identification of COVID-19 cases may assist in triage and infection-control workflows. The high precision and recall observed in both models for normal and bacterial pneumonia categories suggest their potential utility in supporting diagnostic screening in settings with limited radiologic expertise. The statistical equivalence of both SE-enhanced architectures, as demonstrated by McNemar's test, further supports their potential interchangeability in clinical applications, allowing institutions to select models based on implementation feasibility rather than performance concerns.

Despite strong classification performance, several limitations must be acknowledged. First, the dataset used in this study was sourced from a single public repository and may not reflect the full spectrum of radiographic variability seen in real-world clinical populations. No external validation dataset was employed, and the class labels were assumed to be correct based on file naming conventions rather than confirmed by expert radiologists or laboratory data. Further analysis is required to assess the generalizability of these findings across diverse imaging datasets validated by experienced radiologists across real-world multi-institutional clinical environments. Additionally, although the dataset was balanced through stratified sampling, there remains some class imbalance, particularly in the COVID-19 and pneumonia-viral categories, which may affect model generalization. The exclusive use of chest X-rays also omits information from lateral views, which are often clinically informative in diagnosing pneumonia. Lastly, the models were trained and evaluated on relatively small input sizes (224×224 or 299×299 pixels), which, while computationally efficient, may result in loss of fine-grained image detail.

Future work should focus on expanding model evaluation to external datasets, incorporating multi-view and multi-modal inputs (e.g., clinical history, vital signs), and experimenting with other attention mechanisms beyond SE blocks. Exploring hybrid ensemble models or transformer-based vision architectures could also offer performance improvements. Importantly, any model intended for clinical deployment should undergo rigorous validation in diverse populations, with prospective trials comparing its performance to that of human clinicians.

## Conclusions

This study demonstrates the effectiveness of SE-enhanced CNNs, ResNet50V2 and InceptionV3, for multi-class classification of chest X-rays into normal, bacterial-pneumonia, viral-pneumonia, and COVID-19 categories. Both architectures achieved high diagnostic accuracy (97-98%) and AUCs above 0.99, with statistical analysis confirming equivalent performance between models. McNemar's test revealed no significant difference in classification accuracy (χ²=0.1250, p>0.05), indicating that the observed 0.32% accuracy difference between ResNet50V2-SE and InceptionV3-SE reflects natural variance rather than a meaningful performance advantage. Despite this strong performance, both models encountered mild challenges in distinguishing between viral-pneumonia and COVID-19 cases, underscoring the inherent limitations of using PA chest radiographs alone for differentiating between visually overlapping conditions.

These findings suggest that SE-enhanced deep learning models can potentially serve as valuable and interchangeable diagnostic aids, particularly in screening for bacterial and viral pneumonia or identifying normal radiographs in resource-limited settings. The statistical equivalence of both architectures provides institutions with flexibility to select models based on computational resources, deployment infrastructure, or inference speed requirements rather than performance concerns. However, further validation is required to confirm generalizability across diverse populations, imaging protocols, and clinical environments. Future studies should incorporate external datasets, lateral views, and clinical metadata to enhance real-world utility. With additional refinement and prospective validation, SE-augmented CNNs hold promise as assistive tools in radiographic pneumonia triage, antimicrobial stewardship, and resource-conscious workflows.

## References

[1] GBD 2021 Lower Respiratory Infections and Antimicrobial Resistance Collaborators (2024). Global, regional, and national incidence and mortality burden of non-COVID-19 lower respiratory infections and aetiologies, 1990-2021: a systematic analysis from the Global Burden of Disease Study 2021. Lancet Infect Dis.

[2] (2025). Centers for Disease Control and Prevention: multiple cause of death, 1999-2023 results. https://wonder.cdc.gov/controller/datarequest/D158.

[3] Ruuskanen O, Lahti E, Jennings LC, Murdoch DR (2011). Viral pneumonia. Lancet.

[4] Hopstaken RM, Witbraad T, van Engelshoven JM, Dinant GJ (2004). Inter-observer variation in the interpretation of chest radiographs for pneumonia in community-acquired lower respiratory tract infections. Clin Radiol.

[5] Huang KA, Alkadri A, Prakash N (2025). Employing squeeze-and-excitation architecture in a fine-tuned convolutional neural network for magnetic resonance imaging tumor classification. Cureus.

[6] Huang KA, Venkitasubramony V, Prakash NS (2025). Leveraging transfer learning and attention mechanisms for a computed tomography lung cancer classification model. Cureus.

[7] Huang KA, Prakash N (2025). Evaluating the impact of attention mechanisms on a fine-tuned neural network for magnetic resonance imaging tumor classification: a comparative analysis. Cureus.

[8] Rahman T, Chowdhury MEH, Khandakar A (2020). Transfer learning with deep convolutional neural network (CNN) for pneumonia detection using chest X‑ray. Appl Sci.

[9] Ibrahim AU, Ozsoz M, Serte S, Al-Turjman F, Yakoi PS (2021). Pneumonia classification using deep learning from chest x-ray images during COVID-19. Cognit Comput.

[10] Das R, Nayak DSK, Rout CP, Jena L, Swarnkar T (2024). Deep learning techniques for identification of pneumonia: a CNN approach. International Conference on Advancements in Smart, Secure and Intelligent Computing (ASSIC).

[11] Oh Y, Park S, Ye JC (2020). Deep learning COVID-19 features on CXR using limited training data sets. IEEE Trans Med Imaging.

[12] Rajpurkar P, Irvin J, Ball RL (2018). Deep learning for chest radiograph diagnosis: a retrospective comparison of the CheXNeXt algorithm to practicing radiologists. PLoS Med.

[13] Saboo YS, Kapse S, Prasanna P (2023). Convolutional neural networks (CNNs) for pneumonia classification on pediatric chest radiographs. Cureus.

[14] Mujahid M, Rustam F, Álvarez R, Luis Vidal Mazón J, Díez IT, Ashraf I (2022). Pneumonia classification from X-ray images with Inception-V3 and convolutional neural network. Diagnostics.

[15] Prabakaran J, Selvaraj P (2023). Implementation of ResNet-50 with the skip connection principle in transfer learning models for lung disease prediction. Intelligent Systems and Sustainable Computing. ICISSC 2022. Smart Innovation, Systems and Technologies.

[16] Yang X (2020). An overview of the attention mechanisms in computer vision. J Phys Conf Ser.

[17] Hu J, Shen L, Sun G (2018). Squeeze-and-excitation networks. 2018 IEEE/CVF Conference on Computer Vision and Pattern Recognition.

